# The effect of unilateral adrenalectomy on patients with primary bilateral macronodular adrenal hyperplasia

**DOI:** 10.1007/s42000-023-00428-8

**Published:** 2023-02-17

**Authors:** Zhongwei Yu, Jie Gao, Fukang Sun

**Affiliations:** 1 Department of Urology, RuiJin Hospital Lu Wan Branch, Shanghai Jiaotong University School of Medicine, Shanghai, 200020 China; 2 Department of Endocrinology, Putuo Hospital, Shanghai University of Traditional Chinese Medicine, Shanghai, 200062 China; 3 Department of Urology, Ruijin Hospital, Shanghai Jiao Tong University School of Medicine, Shanghai, China

**Keywords:** Primary bilateral macronodular adrenal hyperplasia (PBMAH), Cushing’s syndrome, Unilateral adrenalectomy, Urinary free cortisol

## Abstract

**Purpose:**

To evaluate the long-term effect of unilateral adrenalectomy (uADX) on patients with primary bilateral macronodular adrenal hyperplasia (PBMAH).

**Methods:**

We retrospectively reviewed 29 patients (including 11 men and 18 women) with PBMAH and Cushing’s syndrome (CS) between 2005 and 2019 who underwent uADX in our center. Clinical symptoms, serum cortisol (8:00 a.m., 4:00 p.m., and 0:00 a.m.), 24 h urinary free cortisol (UFC), computed tomography (CT) scan of the adrenal gland, and pituitary nuclear magnetic resonance (MR) scan performed before and after operation were analyzed.

**Results:**

The median follow-up time was 39 (13–134) months. uADX decreased significantly at 24 h UFC (median: 357.14 vs. 89.50 ug/24 h, *P* < 0.001) and serum cortisol (8:00 a.m.) (median: 22.88 vs. 12.50 ug/uL, *P* < 0.001) 1 year after surgery. In total, 17 of 29 patients had normal UFC again 1 year after surgery, while one of them suffered a relapse after 61 months. However, uADX failed to decrease UFC to the normal range in the other patients. Ten of the remaining 12 uncured patients and the relapsed patient finally underwent contralateral adrenalectomy (cADX). The 24 h UFC of the patients who were cured (*n* = 17) after uADX was significantly lower than that of the uncured patients (*n* = 12) (222.30 vs. 579.10 ug/24 h, *P* = 0.011).

**Conclusion:**

uADX may be an appropriate treatment for patients with mildly elevated cortisol, while contralateral adrenalectomy (cADX) may be required for patients with highly elevated cortisol. The level of 24 h UFC is helpful to predict patients’ prognosis.

## Introduction

Primary bilateral macronodular adrenal hyperplasia (PBMAH) is an infrequent disease with characteristics of bilateral adrenal cortex macronodular hyperplasia (nodules are larger than 10 mm and frequently reach 30 to 40 mm in diameter). PBMAH can lead to CS due to overproduction of cortisol due to hyperplastic adrenal tissue, this accounting, however, for no more than 2% of endogenous CS cases [1]. PBMAH is usually detected by radiological findings in those with subclinical CS and overt CS. PBMAH affects women more often than men, with a female-to-male ratio of 2–3:1. Resistant hypertension, hyperglycemia, and hypokalemia symptoms, which are caused by severe CS, make PBMAH a serious threat for patients. The causes of PBMAH have not as yet been definitively identified. Surgical therapy, including treatment of unilateral or bilateral adrenalectomy, remains the mainstay of treatment for PBMAH. Unilateral adrenalectomy (uADX) has, in recent years, tended to be a preferred choice for surgeons to deal with PBMAH so as to avoid Addison’s syndrome caused by bilateral adrenalectomy. However, what are patient outcomes after uADX is still not entirely clarified. Here we report and analyze the follow-up results of 29 cases of PBMAH patients who underwent uADX in our center from 2005 to 2019. We believe that a better understanding of clinical outcomes after uADX will aid in the management of PBMAH.

## Materials and methods

### Study patients

All of the 29 patients enrolled were diagnosed with PBMAH and CS (25 with overt CS and 4 with subclinical CS) by enhanced CT scan of the adrenal glands (bilateral multiple adrenal nodules > 10 mm) and endocrinological examination and verified by postoperative pathologic results. The patients in this study were found to have the following (1) increased midnight serum cortisol or 24 h urinary free cortisol (UFC); (2) suppressed or normal serum adrenocorticotropic hormone (ACTH) (8:00 a.m.); (3) normal sella MR image results; while they were (4) negative to both the low- (2 mg, 2 days) and high-dose (8 mg) dexamethasone suppression test LDDST and HDDST) and their serum cortisol level (reference range 6.7–22.6 ug/dL), UFC (reference range 21–111 ug/24 h), and serum ACTH (reference range 12.00–78.00 pg/mL) were all checked more than twice. All the procedures performed were approved by the institutional review boards of the Ruijin Hospital Ethics Committee. All methods were carried out in accordance with relevant guidelines and regulations.

A total of 29 PBMAH patients (11 men and 18 women) underwent uADX from 2005 to 2019 in our center. Two female patients are sisters from the same family. The others appear to be sporadic cases. The level of serum sex hormone, metanephrine, normetanephrine, and aldosterone in all patients was normal. The age of the patients at diagnosis ranged from 40 to 68 (average 50.8) years old (Table 1). Hypertension (21 cases), moon face, and obesity (three cases), accidental radiologic findings (three cases), and diabetes mellitus (two cases) were the chief complaints of the patients.

**Table 1:** Demographic information and clinical data of patients with PBMAH

Patient no	Sex	Age (years)	BMI (kg/m^2^)	Diabetes mellitus	Hypertension	Surgery options	uADX site	Adrenal gland volume (cm)	Remaining adrenal gland volume (cm)
P01	F	52	30.44	+	+	Open	R	16.0 × 8.0 × 3.0	7.0 × 5.5 × 5.0
P02	F	53	24.97	−	−	Open	R	8.0 × 6.0 × 4.0	/
P03	M	68	25.47	−	+	Open	R	7.0 × 7.0 × 2.0	5.2 × 4.9 × 3.0
P04	M	52	/	+	+	Open	R	5.0 × 3.5 × 3.0	6.5 × 6.1 × 5.0
P05	M	53	20.7	+	+	Open	R	7.0 × 4.5 × 3.0	6.6 × 5.2 × 3.8
P06	F	42	/	−	+	Open	R	6.0 × 5.0 × 4.0	8.2 × 4.5 × 3.0
P07	M	57	20.66	−	+	Open	R	7.0 × 6.0 × 5.0	4.0 × 2.7 × 2.4
P08	M	45	24.8	−	+	Open	R	7.5 × 3.0 × 2.5	8.0 × 7.5 × 3.3
P09	M	49	25.08	+	+	Open	R	8.0 × 7.0 × 3.0	7.0 × 6.0 × 5.7
P10	F	40	26.95	+	+	Open	R	9.0 × 6.0 × 3.0	9.0 × 4.8 × 4.4
P11	F	62	26.84	+	+	Open	R	9.0 × 7.0 × 2.0	7.0 × 4.9 × 4.0
P12	F	46	22.67	+	+	Laparoscopic	R	6.0 × 5.0 × 4.0	4.5 × 4.0 × 2.0
P13	F	49	21.48	+	+	Laparoscopic	L	9.5 × 4.5 × 3.3	6.0 × 5.4 × 2.3
P14	F	56	26.63	+	−	Laparoscopic	L	8.0 × 7.0 × 2.0	4.0 × 4.0 × 2.5
P15	F	60	28.57	+	+	Laparoscopic	R	8.5 × 5.0 × 2.0	5.5 × 5.3 × 2.3
P16	M	50	27.40	+	+	Laparoscopic	R	11.0 × 6.0 × 3.0	7.8 × 4.0 × 3.0
P17	F	45	19.13	−	+	Laparoscopic	L	8.0 × 7.0 × 2.5	6.0 × 3.6 × 3.0
P18	M	50	19.82	−	+	Laparoscopic	L	10.0 × 5.5 × 1.5	6.5 × 5.0 × 3.0
P19	F	61	22.89	+	+	Laparoscopic	R	10.0 × 4.0 × 2.0	6.0 × 4.0 × 3.0
P20	F	53	/	−	+	Robotic	L	7.0 × 6.0 × 2.0	6.0 × 4.0 × 2.5
P21	F	49	26.72	−	+	Laparoscopic	L	9.0 × 4.0 × 3.0	5.0 × 4.0 × 3.0
P22	F	58	27.22	+	+	Laparoscopic	L	7.5 × 5.0 × 3.5	5.0 × 3.5 × 2.5
P23	F	46	25.25	−	+	Laparoscopic	L	5.0 × 3.0 × 2.0	4.0 × 2.5 × 2.0
P24	F	52	24.90	−	+	Laparoscopic	L	8.0 × 6.0 × 2.5	6.0 × 4.5 × 4.0
P25	M	45	/	−	+	Laparoscopic	L	11.0 × 6.0 × 1.5	7.0 × 6.0 × 2.5
P26	F	43	26.81	−	+	Laparoscopic	R	7.0 × 6.5 × 3.5	6.0 × 5.0 × 2.0
P27	M	42	26.56	+	−	Open	L	6.0 × 5.0 × 3.0	5.0 × 3.7 × 3.0
P28	F	45	27.10	−	+	Robotic	L	4.0 × 3.0 × 2.0	3.5 × 2.2 × 1.5
P29	M	51	24.86	−	−	Laparoscopic	R	4.0 × 4.0 × 2.8	4.5 × 4.0 × 2.2

*PBMAH*, primary bilateral macronodular adrenal hyperplasia; *M*, male; *F*, female; *BMI*, body mass index; *uADX*, unilateral adrenalectomy; + , positive; − , negative; /, data missing; *open*, open surgery; *laparoscopic*, laparoscopic surgery; *robotic*, robotic assistant laparoscopic surgery; *R*, right side adrenal gland; *L*, left side adrenal gland

### Treatment

All patients were prepared for surgery and underwent uADX of the larger gland (17 right and 12 left glands) by experienced urologists in our center. Twelve patients had open uADX (11 right glands and one left). Fifteen patients underwent laparoscopic uADX (six right and nine left glands). Two patients underwent robotic assistant laparoscopic uADX (both left glands). The volume of the biggest mass we successfully excised was 16.0 × 8.0 × 3.0 cm.

### Follow-up

Patients were advised to undergo an abdominal CT imaging scan and UFC (reference range 21–111 ug/24 h), serum ACTH (reference range 12.00–78.00 pg/mL), and serum cortisol concentration test at 0:00 a.m., 8:00 a.m., and 4:00 p.m. (reference range 6.7–22.6 ug/dL) after the uADX. These patients were scheduled to consult the urologist in 1, 3, 6, and 12 months postoperatively, and thereafter, to have a once or twice-a-year follow-up, according to each patient’s recovery rate. The median follow-up time was 39 months (range: 13–134).

Remission was defined as postoperative normal UFC and serum cortisol or the occurrence of adrenal insufficiency, while recurrence was defined as a UFC level above the normal value again in a patient who was previously in remission. Patients who were not in remission 1 year after uADX were advised to undergo contralateral adrenalectomy (cADX). Patients who underwent cADX were subsequently treated with cortisol replacement therapy throughout their lives and underwent a serum ACTH or serum cortisol test. To assess the risk factors and prognosis, we divided the patients into remission and non-remission groups and compared serum cortisol (8:00 a.m.), UFC, BMI, and blood pressure between the two groups.

### Statistical analysis

SPSS 22.0 was applied to process the clinical data. The effects of uADX were assessed with Wilcoxon’s nonparametric signed-rank test (UFC) and the paired-sample *t*-test (serum cortisol and ACTH). An analysis of the preoperative differences between the patients in the remission and the non-remission group was postoperatively assessed with the Mann–Whitney U test (including BMI and UFC) and the independent samples *t*-test (including age, serum cortisol, ACTH, and blood pressure). We used the chi-square test to compare qualitative variables. Results are expressed as median (range). *P* < 0.05 means that the difference is statistically significant.

## Results

### UFC, ACTH, and serum cortisol before and 1 year after surgery

The median UFC was 357.14 (range: 108.00–1361.80 ug) ug/24 h before uADX, compared to 89.50 (range: 38.22–440.82) ug/24 h 1 year after uADX (*P* < 0.001). Postoperative 8:00 a.m. ACTH levels showed a significant elevation compared with preoperative ACTH: preoperative, 7.91 pg/mL (range: 3.23–14.60) vs. postoperative, 11.70 pg/mL (range: 3.19–20.02) (*P* = 0.025). Serum cortisol (8:00 a.m.) decreased from 22.88 (range: 10.32–39.24) ug/dL to 12.50 (range: 5.66–37.80) ug/dL (*P* < 0.001) after uADX. Serum cortisol (4:00 p.m.) decreased from 18.59 (range: 5.55–33.64) ug/dL to 8.62 (range: 3.68–31.78) ug/dL (*P* < 0.001) after uADX. Serum cortisol (0:00 a.m.) decreased from 17.90 (range: 3.85–29.60) ug/dL to 7.84 (range: 2.36–23.82) ug/dL (*P* < 0.001) after uADX (Table 2).

**Table 2:** Preoperative and 1-year postoperative UFC, ACTH, and serum cortisol of patients treated with uADX

Patient no	UFC (ug/24 h)		ACTH (8:00 a.m.) (pg/mL)		Serum cortisol (8:00 a.m.) (ug/dL)	
	Before	After	Before	After	Before	After
P01	1212.00	238.66	5.20	3.85	34.78	11.30
P02	543.10	277.40	/	/	26.6	13.00
P03	340.00	53.30	14.60	15.1	17.3	8.50
P04	184.30	89.50	5.40	6.32	14.8	10.00
P05	557.10	70.00	8.01	7.07	27.4	13.79
P06	736.00	173.60	9.73	9.42	32.9	15.33
P07	240.15	78.76	8.73	20.02	28.02	9.36
P08	222.30	72.90	10.20	12.53	18.99	9.30
P09	615.10	223.50	7.31	/	25.1	14.77
P10	1361.80	440.82	5.05	13.48	22.88	20.11
P11	406.70	312.23	8.90	6.78	30.45	8.75
P12	128.64	85.40	7.09	13.82	14.78	14.53
P13	390.90	87.48	4.52	16.26	18.40	12.66
P14	131.84	78.10	6.34	16.64	10.32	9.66
P15	143.64	41.08	3.75	11.70	26.46	14.68
P16	540.00	255.15	7.95	7.93	37.80	20.01
P17	357.14	56.76	5.37	/	23.91	5.66
P18	930.60	73.24	8.56	11.80	22.22	12.50
P19	962.40	365.33	6.78	4.43	39.24	20.23
P20	822.13	101.23	10.41	9.07	25.26	20.20
P21	184.80	62.03	7.88	14.13	12.51	12.49
P22	151.20	49.80	5.16	12.43	12.30	9.07
P23	323.40	195.60	11.40	5.43	22.80	21.90
P24	725.44	122.56	13.10	9.20	27.32	10.94
P25	1031.7	107.73	3.23	5.94	29.53	9.57
P26	204.00	134.72	8.15	3.19	15.21	10.97
P27	245.20	130.40	10.40	13.16	21.70	9.46
P28	166.60	67.90	10.63	12.51	14.10	13.11
P29	108.00	50.10	6.84	14.92	12.65	13.30
Median	357.14	89.50	7.91	11.70	22.88	12.50
*P*	< 0.001		= 0.025		< 0.001	

*uADX*, unilateral adrenalectomy; *UFC*, 24 h urinary free cortisol reference range 21–111 ug/24 h; *ACTH*, adrenocorticotropic hormone reference range 12.00–78.00 pg/mL; /, data missing; serum cortisol (8:00 a.m.) reference range (6.7–22.6 ug/dL). The effects of uADX were assessed using Wilcoxon’s nonparametric signed-rank test (UFC) and the paired-sample *t*-test (serum cortisol and ACTH)

### Clinical symptoms

A total of 25 patients presented hypertension before surgery. Blood pressure returned to normal levels after surgery in-five patients without their taking any antihypertensive drugs. A total of 14 patients presented hyperglycemia. Five of them (35.7%) had normal levels of serum glucose again, having ceased taking hypoglycemic drugs after uADX.

### Remission and recurrence

**Fig. 1:**
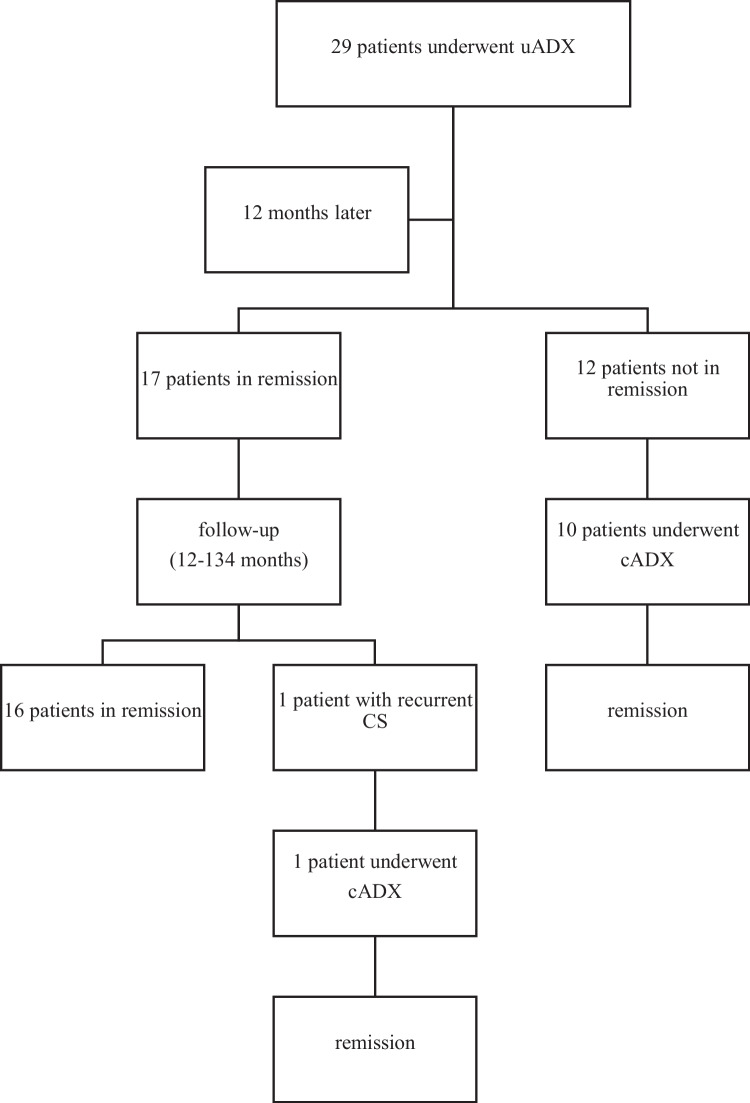
Prognosis of patients with PBMAH after unilateral adrenalectomy. *uADX*, unilateral adrenalectomy; *cADX*, contralateral adrenalectomy; *CS*, Cushing’s syndrome

Seventeen patients (58.6%) had controlled CS returning to normal serum cortisol and UFC levels 12 months postoperatively (Fig. 1). The patients’ median UFC was reduced from 222.30 (range: 108.00–1031.70) to 72.90 (range: 41.08–107.73) ug/24 h 1 year after surgery, *P* < 0.001. The median follow-up time of the patients in the remission group was 33 (range: 13–106) months. The UFC of one patient in the remission group rose again 61 months later. The patient’s contralateral adrenal gland was excised in the 61 months after uADX because of a recurrence of CS.

uADX failed to decrease UFC to normal levels in a total of 12 patients in the first year after the operation. The median UFC of these patients decreased from 579.10 (range: 204.00–1361.80) to 231.08 (range: 122.56–440.82) ug/24 h 1 year after the operation, *P* = 0.002. As a result, ten of them underwent cADX to normalize serum cortisol levels. The other two patients with mildly elevated UFC after surgery were followed up instead of undergoing cADX.

None of the patients suffered an adrenal crisis during the study period.

### Prognostic risk factors

The patients in the non-remission group (*n* = 12) had significantly higher levels of preoperative UFC (579.10 vs. 223.30 ug/24 h, *P* = 0.011) and serum cortisol (8 a.m.) (26.96 vs. 18.40 ug/dL, *P* = 0.002) as well as larger remaining adrenal glands (maximum diameter 6.63 ± 1.43 vs. 5.51 ± 1.21 cm, *P* = 0.035) than the patients in the remission group (*n* = 17) (Table 3). However, sex, age, blood pressure, and BMI were not significantly different between the two groups.

**Table 3:** Comparison of clinical data of patients in remission group and non-remission group before uADX

Variables	Group		*p*
	Remission	Non-remission	
*n*	17	12	-
Male	8	3	0.273
Female	9	9	
Age Median (range), years	51.0 (45–68)	49.5 (40–62)	0.324
UFC Median (range), ug/24 h	222.30 (108.00–1031.70)	579.10 (204.00–1361.80)	0.011
Serum cortisol (8:00 a.m.) Median (range), ug/dL	18.40 (10.32–29.53)	26.96 (15.21–39.24)	0.002
ACTH (8:00 a.m.) Median (range), pg/mL	7.09 (3.23–14.60)	8.15 (5.05–13.10)	0.319
BMI Median (range), kg/m^2^	24.83 (19.13–28.57)	26.56 (22.89–30.44)	0.107
Systolic pressure Median (range), mmHg	160 (130–230)	160 (130–200)	0.538
Diastolic pressure median (range), mmHg	100 (80–140)	100 (70–130)	0.647
Size of the remaining gland Mean ± SD, cm	6.63 ± 1.43	5.51 ± 1.21	0.035

*uADX*, unilateral adrenalectomy; *UFC*, 24 h urinary free cortisol reference range 21–111 ug/24 h; *ACTH*, adrenocorticotropic hormone reference range 12.00–78.00 pg/mL; *BMI*, body mass index; serum cortisol (8:00 a.m.) reference range (6.7–22.6 ug/dL). a Mann–Whitney *U* test (including BMI and UFC), independent-samples *t*-test (including age, serum cortisol, ATCTH, size of the remaining gland, and blood pressure), and chi-square test (gender) were used to analyze data of remission group and non-remission group

## Discussion

PBMAH is a rare but challenging disease that can cause subclinical or overt CS. Overproduction of cortisol due to nodules of the adrenal cortex leads to a series of typical symptoms, including central obesity, arterial hypertension, impaired glucose tolerance, edema, and hypokalemia. Most PBMAH patients present in the 5th and 6th decades, a later age of onset compared with that of unilateral adenomas or CS [2]. Over the past few years, numerous studies have revealed that PBMAH more often has a genetic background than what was once thought [3]. The most important recent finding in this field is the discovery of ARMC5 germline mutations in 50% of patients with apparently sporadic PBMAH [4–6]. The etiology of PBMAH has not yet been entirely elucidated. However, a large amount of research has demonstrated that aberrant expression of various G-protein-coupled receptors (GPCRs), including the β-adrenergic receptor, vasopressin receptor, and luteinizing hormone/choriogonadotropin receptor (LHCGR) in the adrenal cortex plays an important role in PBMAH [7, 8]. Aberrant regulation of GPCR stimulates paracrine ACTH production through PBMAH cells acting on the ACTH receptor (melanocortin-2 receptor, MC2R), which leads to the synthesis of cortisol even if the circulating ACTH level is low [9, 10]. CS is a life-threatening disease and results in 2–4 times increased mortality, mainly due to cardiovascular events [11, 12]. Thus, earlier diagnosis and treatment of hypercortisolism are crucial in order to avoid the comorbidities associated with the disorder.

Because PBMAH almost never progresses to a malignant lesion, the purpose of treatment is to reduce the secretion of cortisol into the blood and normalize tissue exposure to cortisol so as to prevent increased morbidity and mortality. To date, surgery has remained the only way to cure PBMAH. Bilateral adrenalectomy was considered to be the standard treatment of PBMAH in the past [13], and complete excision of bilateral adrenal glands was recommended in order to avoid relapse. Bilateral adrenalectomy can maximally decrease cortisol in the blood and relieve the condition. However, patients require lifelong steroid replacement therapy after surgery due to a lack of cortisol. Moreover, if the steroid replacement therapy is discontinued, life-threatening adrenal insufficiency, known as Addison’s syndrome, can develop rapidly [14]. The latter explains why an increasing number of patients have undergone unilateral adrenalectomy in recent years [15, 16]. In our experience, uADX tends to be the first choice for surgeons to manage PBMAH because of the following advantages. ① uADX decreases the risk of surgical injury compared with bilateral adrenalectomy, while uADX may be a perfect option for elderly PBMAH patients and patients with debility due to cardiopulmonary dysfunction. ② PBMAH has been proven to develop slowly, while uADX can relieve CS for a long time in patients with mildly elevated cortisol. ③ uADX as staged treatment may reduce mortality caused by adrenal crisis, especially in patients with severe CS due to residual adrenal function. ④.Patients will avoid cortisol replacement treatment if they have a favorable prognosis after uADX.

Laparoscopic procedures are safe, effective, and less invasive than open adrenalectomy [17]. Although urologists tend to prefer uADX in patients with PBMAH, in fact, the procedure sometimes does not relieve the condition, particularly in patients with high levels of preoperative UFC and serum cortisol. In our study, we found that 58.6% of CS patients (17 of 29 patients) were in remission 1 year after uADX, while there were still 12 patients with high UFC 1 year after surgery. In another study, at the last follow-up (median, 50 months), only 32% of the patients with unilat-ADX-PBMAH were biochemically controlled [18]. Furthermore, the patients in the non-remission group had higher UFC and serum cortisol (8:00 a.m.) levels than the patients in the remission group. Hence, urologists should have a plan for cADX in mind before conducting uADX, in particular for patients with high UFC. Interestingly, recurrence was seen to be uncommon in patients after uADX. Only one of 17 patients suffered a relapse in our study. This is partially because the initial UFC of these patients was lower than that of the non-remission patients, which can mean that the smallest adrenal gland (the residual one after surgery) did not present glucocorticoid hypersecretion.

Our study indicates that patients with high levels of cortisol (serum cortisol and UFC) and the larger remaining gland are more likely to need cADX. Our results are consistent with those previously reported. In patients with PBMAH and mildly increased cortisol production (UFC less than twice the upper limit of normal), uADX might be more effective [19]. High levels of cortisol implies more active secretion by bilateral adrenal glands and that even the remnant single adrenal gland remaining after uADX can still cause CS. Thus, we can infer the prognosis of patients with PBMAH according to their initial UFC and serum cortisol as follows: patients with mildly elevated UFC appear to achieve long-term remission after uADX, while cADX for patients with abnormally high UFC is usually inevitable.

PBMAH is known to be a rare disease that still involves many challenges with regard to its diagnosis and treatment. Our study has sought to clearly and comprehensively present the prognosis of postoperative patients with PBMAH. However, due to the retrospective nature of our study, some clinical data may be lacking or incomplete.

Because uADX is not a definitive cure, it is crucial for patients with PBMAH to keep in touch with their doctor and have a physical examination regularly long-term. Patients in remission after uADX can decrease the frequency of follow-up since recurrence was not observed to be common in patients after uADX. The serum cortisol and UFC test is a good marker to evaluate disease, while an enhanced CT scan of the adrenal gland aids in determining whether there is residual adrenal tissue at the site of the surgery or if the contralateral gland is continuing to grow.

## Conclusions

uADX may be an appropriate treatment for patients with mildly elevated cortisol (including serum cortisol and 24 h UFC). If a uADX is performed in the case of PBMAH, the physician must weigh the risks of having to perform a cADX later in the course of the condition. A unilateral procedure might not yield a cure, while bilateral adrenalectomy carries the unavoidable risk of adrenal insufficiency. Therefore, uADX constitutes better treatment for patients with PBMAH and CS.

## Data Availability

The data analyzed during the current study are available from the corresponding author on reasonable request.
